# Pregnancy Rates in Horro and Holstein-Horro Cross-Breed Cattle Breeds Following AI With Sexed Semen Under the Smallholder Farming System in Central Ethiopia

**DOI:** 10.1155/vmi/5435098

**Published:** 2025-05-03

**Authors:** Afework Legese Endebu, Mohammed Aliy Deresh, Aregaw Abera Dodicho

**Affiliations:** ^1^Ameya District Agricultural Office, South West Shoa Zone, Amaya, Oromia, Ethiopia; ^2^Department of Animal Sciences, College of Agriculture and Veterinary Medicine, Jimma University, P.O. Box 307, Jimma, Ethiopia

**Keywords:** breed, pregnancy rate, semen type, smallholder farming

## Abstract

This study aimed to evaluate the pregnancy rate achieved with sexed semen (SS) in Horro and Holstein-Horro cross-breed cattle under a smallholder farming system in Central Ethiopia. 120 cows and heifers (60 Horro and 60 Holstein-Horro cross-breed) were enrolled in the study. Selection criteria included parity, body condition score (BCS), and pregnancy status of the animals. To synchronize estrus, all selected animals received a single injection of prostaglandin (PGF2α). Estrus signs were monitored to assess heat response and the time to estrus induction in the treated animals. The responded animals were then randomly inseminated with either SS or conventional semen (CS). The conception rate was calculated based on the number of animals that exhibited estrus, were inseminated, and subsequently conceived. Data analysis was conducted using SAS (Version 9.4). Estrus response and conception rate data were analyzed using logistic regression while the interval to estrus was analyzed using a general linear model. Out of the 120 synchronized animals, 44 Horro and 52 Holstein-Horro cross-breed cows and heifers responded to PGF2α, resulting in an estrus response rate of 80%. Body condition and parity significantly influenced estrus response (*p* < 0.05). The breed had a significant effect (*p* < 0.05) on the interval from PGF2α injection to the onset of estrus, while parity and BCS did not show significant effects. Upon pregnancy detection, 56.8% of Horro and 63.5% of Holstein-Horro cross-breed cows and heifers were found to be pregnant; among these, 65.1% were cows and 56.6% were heifers across both semen types. The type of semen used had a significant impact on the pregnancy rate (*p* < 0.05), with a pregnancy rate of 72.9% for CS compared to 47.9% for SS. Overall, the pregnancy rate of 47.9% achieved with SS is promising and exceeds the national pregnancy rate for first inseminations using CS, which ranges from 7.14% to 40.23%.

## 1. Introduction

Agriculture in Ethiopia is an important economic sector upon which most Ethiopians depend for food, feed, and income [1]. The sector contributes about 50% to the overall gross domestic product (GDP), generates 90% of export earnings, and employs 80% of the population [2]. Livestock plays a crucial role in Ethiopian agriculture, contributing to 45% of the overall value of agricultural production, 19% of the GDP, and generating 16%–19% of the country's foreign exchange earnings. Additionally, it supports the livelihoods of approximately 60%–70% of the population [3]. The country has significant livestock resources and holds the largest livestock population in Africa, estimated at around 70.29 million cattle [4]. The local cattle breeds comprise 97.4% of the total, while cross breed and exotic breeds represent approximately 2.29% and 0.31%, respectively [3]. Despite the large number of indigenous breeds, the productivity of the local cattle breed is low due to the low genetic potential of the indigenous breeds, low level of input, high disease prevalence and poor animal health services, and low level of husbandry practice [5, 6]. In contrast, there is an increasing demand for livestock products, particularly milk and meat, due to the increasing human population, urbanization trends, and rising household incomes [7].

In Ethiopia, a significant barrier to livestock productivity, particularly in the dairy sector, is the lack of access to adaptable, high-yielding improved genetics. Despite a strong demand for better breeds, the country lacks a multiplication center to provide improved heifers. The primary source of cross-breed heifers comes from artificial insemination (AI) services, which are mainly offered by public institutions and a few private entities [8]. The challenges are more confounded by poor AI service efficiency due to conception failure, heat detection problems, and improper timing of insemination [9]. Therefore, fulfilling the growing demand for milk and dairy products is unattainable without a significant increase in the number of high-producing, locally adapted cows and enhancements in productivity per cow [10]. Assisted reproductive technologies have proven to be transformative in altering dairy herd composition and boosting milk production. Among these technologies, sexed semen (SS) stands out as an innovative biotechnological tool that improves production efficiency, reproductive performance, and the economic viability of dairy farms by increasing the birth rate of genetically superior heifers following AI [11, 12]. Utilizing semen that is enriched with sperm capable of producing offspring of a specific sex can be an effective strategy for producers to gain better control over production results. This approach not only helps to address welfare concerns associated with the premature death of unwanted animals but also serves as a potential support in navigating stricter animal welfare regulations [13].

Even with its benefits, the adoption of the technology is limited to commercial dairy herds in developed countries. Among the factors hindering the applicability of SS in smallholder dairy production systems in developing countries like Ethiopia are its low conception rate and high prices [14]. The lower pregnancy rates per AI (P/AI) using SS as compared to conventional semen (CS) are mainly attributed to the low sperm dose per insemination and reductions in sperm quality and viability caused by damage during the sorting process [15]. Other factors such as herd size [16], breeding season and estrus detection type [17], climatic regions [18], heifer age at breeding [19], temperature and humidity surrounding breeding, and AI technician [20] are also affecting the pregnancy rate using SS. As a result, the average pregnancy rates are reduced to 21%–65% in SS as compared to CS [18–23]. The breed is another factor that affects P/AI even using CS. Previous study reports indicated that crossbreeds have a higher P/AI than local breeds [24, 25]. Using SS, Dawod and Elbaz [26] and Bittante et al. [27] also obtained a higher odds ratio of conception in the Simmental cows than the Holstein-Friesian cows. Furthermore, the parity number is another factor associated with differences in P/AI. As anticipated, virgin heifers are more fertile than lactating dairy cows, as a result of not having the contemporary metabolic burden of milk production [27–29]. Despite the effect of the abovementioned factors on the pregnancy rate, the combined effects of breed and parity on the conception rates of dairy cattle inseminated with SS in smallholder farming contexts where a significant portion of the country's milk production occurs have not been adequately addressed. The Ameya district is a key region for milk production in the country. However, the pregnancy rates following AI with SS in smallholder farming systems in this area have received little assessment, and their significance remains poorly understood and undocumented. Therefore, to gain a better insight into the effectiveness of SS, it is essential to monitor its performance to develop suitable recommendations for enhancing the dairy cattle breeding program in the country. Therefore, this research aimed to evaluate the pregnancy rates of Horro and Holstein-Horro cross-breed cattle inseminated with SS under a smallholder farming system.

## 2. Materials and Methods

### 2.1. Description of the Study Area

The study was carried out at Ameya district South West Shoa Zone, Oromia region. The district is located 144 km southwest of Addis Ababa with altitude ranging from 1850 to 2800 m above sea level. The area is part of the central highlands of Ethiopia. The average annual minimum and maximum temperatures were 15° and 24°C, respectively. The annual rainfall ranged from 1350 to 1600 mm. The area receives bimodal rainfall with two rainy seasons in a year.

### 2.2. Experimental Animals and Their Management

All animal handling and experimental procedures were approved by the Animal Care and Use Committee of the Jimma University College of Agriculture and Veterinary Medicine.

The study animals were selected from different dairy farms. Dairy herds were selected based on their uniformity in the management system and the willingness of the farmer to participate. Accordingly, a total of 120 dairy cattle, 60 Horro (30 cows and 30 heifers) and 60 Holstein-Horro cross-breed (30 cows and 30 heifers), were enrolled in the study. All cows were greater than 60 days postpartum and in parities 1–4. The study animals were selected based on selection criteria such as overall health, presence of mature corpus luteum, absence of pregnancy, and body condition score (BCS). The BCS ranged from 2 to 4 on a scale of 1 to 5. Rating the BCS was done subjectively based on the fat cover and flesh over the ribs, loin, and tail head [30]; BCSs were evaluated through optimal when ≥ 3 BCS and suboptimal when < 3 BCS [31]. The reproduction status of cows and heifers was confirmed based on the information from the owners and through internal reproductive organ palpation. The selected animal's reproductive organs were palpated per rectum to assess pregnancy and the presence of corpus luteum on either of the ovaries.

### 2.3. Study Design

Candidate animals were divided into two by breed (Horro and Holstein-Horro cross-breed) and within breed by parity (cows and heifers). During the commencement, all animals that have a CL on either of their ovaries were injected 2 mL of PGF2α (Synchromate, Bremer Pharma GMBH, Germany, 1 mL solution of Synchromate containing cloprostenol 0.263 mg equal to cloprostenol 0.250 mg) intramuscular. The animals that received the hormone were closely observed for any sign of estrus for five consecutive days. Any signs of estrus (restlessness, redness of vulva, mounting others, standing to be mounted, vaginal mucus discharge, frequent urination, sniffing, bellowing, and other related signs) were recorded based on visual observation and information from the owners. Animals that exhibited estrus were randomly allocated to be inseminated by SS or CS. Insemination was done 12–18 h after the onset of estrus. The number of pregnant cows and heifers following AI was determined by the number of animals that exhibited estrus and were subsequently inseminated. Pregnancy diagnosis was conducted by rectal palpation 60 days after AI.

### 2.4. Statistical Analysis

Statistical analyses were performed with SAS (Version 9.4, SAS Institute Inc., Cary, NC). Estrus expression and conception rate data were analyzed using binary logistic regression while data on the interval to estrus were analyzed using a general linear model, and the significance level was set at *p* < 0.05.

## 3. Results and Discussion

### 3.1. Estrus Response to PGF2α Hormone

The results of the estrus response rate to PGF2α hormone in the study area are presented in Table 1. When PGF2α is administered to animals with a functionally mature corpus luteum, 85% to 95% are expected to reach estrus within 7 days of treatment, and 70% to 90% show estrus signs 3 to 5 days after treatment [32]. In this study, 80% (96/120) of animals responded to the PGF2α hormone treatment. This consisted of 19 Horro cows and 25 heifers and 24 Holstein-Horro crossed cows and 28 heifers. The logistic regression analysis indicates parity's significant effects on the estrus response rate. As a result, heifers were 2.99 times more likely to respond to the treatment than cows (OR = 2.99; 95% CI: 1.13–7.87). This indicates that the efficacy of the treatment regime is better in heifers than in cows in the study area. This might be associated with differences in their reproductive physiology, differences in body condition, and the presence of functional corpus luteum at the time of injection. Additionally, it could be due to the management system in general and the feeding system in particular. In the highlands of Ethiopia, smallholder dairy farmers feed their animals mainly natural pasture, crop residues, and nonconventional feeds [33, 34] which are generally poor in nutritional value. As a result, if no compensatory intake of nutrients is achieved to cope with the requirement for multiparous cows, reproductive functions, including hormone synthesis and secretion, as well as follicle ovulation may be depressed and thereby reduces the response in the overall reproductive performance [35, 36]. Higher estrus responses in the nulliparous females in this study corroborate these earlier findings of Chanyalew et al. [37], and Tadesse et al. [38] obtained higher estrus response in heifers as compared to cows in similar management systems; also, Bonato et al. [36] obtained a lower rate of estrus in primiparous (61.36%) compared to nulliparous (76.39%) females in Nelore cattle. In contrast, Haile et al. [24] reported a higher proportion of estrus response in multiparous cows as compared to heifers using a similar approach in a similar management system. Diaz et al. [39] also obtained similar results in Brown Swiss cattle. The differences could be attributed to various factors such as the management systems used by dairy farmers before the estrus synchronization protocol, the genetic factors of the animals, and the type and efficacy of drugs used compared to other studies.

Even though there was no breed effect on estrus response (*p* > 0.05), numerically higher proportion (86.67%) of estrus response was recorded in crossbreds compared to local breeds (73.33%) in those who received a similar dose of hormone injection. This may be attributed to variations in hypothalamic, pituitary, and ovarian functions between Zebu and *Bos taurus* cattle, resulting in differing fertility levels between the two species, even when subjected to the same feeding conditions [40, 41]. Similarly, Haile et al. [24] observed a higher estrus response rate (100%) in cross-breed animals than local (86.7%). Gugssa et al. [42] and Tadesse et al. [38] also reported similar results. In the current study, the BCS significantly influenced (*p*=0.001) the estrus response rate among the animals that received the hormone injection. Those suboptimal BCSs were 0.17 times less likely to respond to the hormone than their counterparts (OR = 0.17; 95% CI: 0.06–0.47). This is in line with the fact that greater BCS and positive energy balance caused greater estrus expression [43]. BCS is associated with ovary functions, and animals with higher BCS produce larger dominant follicle size and improve ovulation rate, and thereby estrus response rate [44, 45]. Quintana-Utra et al. (2019) found that a significant percentage of cows in the group with a BCS lower than 2.5 exhibited conditions compatible with anestrus, indicating a state of reproductive inactivity characterized by a lack of cyclical activity and reduced ovarian function. They associated it with nutritional deficiencies (proteins, vitamins, minerals, and lipids) due to its relation with body condition. This reflects the situation in Ethiopia, particularly the smallholder farming system, where undernutrition or inadequate intake of nutrients relative to metabolic demands is a major factor hindering livestock production systems, particularly among animals dependent upon natural forages for most or all of their feed requirements [47, 48]. The result of the current study is in agreement with the report of Kebede et al. [49] who also obtained greater estrus response in cows and heifers with optimal BCS than those with suboptimal BCS. Richardson et al. [50] also noted a decreased expression of estrus in under-conditioned animals compared to animals in moderate conditions.

### 3.2. Time Interval to Estrus After PGF2α Injection

Regardless of whether cows are inseminated during natural or synchronized estrus, the timing of AI is determined by the onset of estrus, based on the assumption that the interval between the start of estrus and ovulation remains consistent across animals. Inaccurate timing of insemination in relation to estrus onset and ovulation is one of the key factors that limit optimal conception rates in cattle breeding, especially in smallholder farming systems where record-keeping is often lacking. Hence, once a response to PGF2α is observed, understanding the timing of estrus onset becomes crucial, as the interval between the onset of estrus and ovulation can significantly influence the success of AI [51]. The results of the interval to estrus after PGF2α injection in the study area are presented in Table 2. In this study, the interval from PGF2α injection to the onset of estrus was significantly affected by breed (*p* < 0.05), while the effect of parity and BCS was not significant. Regarding the breed, a longer time interval was observed in Horro than in Holstein-Horro crossed animals. The interval to estrus after PGF2α depends on the stage of the estrous cycle at the time PGF2α is administered [52]. PGF2α is only effective in diestrus animals meaning it is only effective in the presence of a functional CL from Day 7 to Day 17 of a normal estrous cycle (estrus = Day 0) [53]. As a result, PGF2α administration in midcycle (Day 8 to Day 11) or later in the luteal phase (Day 12 to Day 15) results in a mean time to estrus of 70 and 62 h, respectively [32]. In general, while luteolysis can be effectively synchronized by administering PGF2α during the diestrus period, the onset of behavioral estrus may occur anywhere from 2 to 5 days later. This variability is attributed to different factors such as the estrous cycle stage at the time of PGF2α treatment [32], differences in the size of the dominant ovarian follicle at the time of injection [54], the time required for an ovulatory follicle to develop [55], dosage of PGF2α injected [56], differences among cows in the rate of regression of the CL following treatment [52], and nutrition and environment [57]. Breed is another factor influencing the time interval from PGF2α injection to the onset of heat [58, 59]. The delayed onset of estrus following PGF2α in local breeds as compared to their counterparts could be associated with hormonal factors such as differential response to PGF2α between *B*. *taurus* and *B. indicus* [60], reduced sensitivity to PGF2 α in luteal tissue in *B. indicus* cattle compared with *B*. *taurus* [59, 61], and amount of released estrogen and time response to estrogen hormone [62]. This study produced results that corroborate the findings of a great deal of the previous work in this field. Gugssa et al. [42] found that 50% of the animals exhibited estrus within 72–96 h, while 40% within 48–72 h, regardless of breed and parity. Demis et al. [63] and Ejigayehu and Alemayehu [64] also reported a similar range of 77.82 ± 2.74 h and 74.53 ± 5.16 h, respectively, in identical management systems. The reports of Dodicho et al. [65] were also in close range (62.4 ± 4.59 h) for cows using a single shot of PGF2α. In the same study, however, heifers responded in shorter intervals (54.35 ± 4.34 h) than heifers in this study (71.75 ± 2.3 h). Chanyalew et al. [37] also recorded that most of the animals come to estrus for more than 96 h.

### 3.3. Pregnancy Rate to Sexed and CS

The results of the pregnancy rate of PGF2α hormone–treated animals in the study area are presented in Table 3. In this study, out of 96 Horro and Holstein-Horro cross-breed cows and heifers that responded to PGF2α, 58 animals (60.4%) became pregnant. Out of these pregnant animals, 25 of them were Horro cows and heifers and the remaining 33 were Holstein-Horro crossed cows and heifers. Thus, the overall pregnancy rate was 60.4%. Except for semen type, the others including breed, parity, and BCS did not influence the pregnancy rate in the current study. The pregnancy rate was significantly higher in using CS (35/48 = 72.9%) than in SS (23/48 = 47.9%). In general, P/AI in animals inseminated with CS has been found greater than that of animals inseminated with SS. This could be due to fewer numbers of sorted sperm per straw [66], the process of sperm sorting often leads to some damage to sperm structure [67], and reduced lifespan of SS in the female reproductive tract [13]. The present findings seem to be consistent with other research which found P/AI using SS at first service 47% for Holstein heifers and 53% for Jersey heifers [68], overall pregnancy rate of 51% for Holstein-Friesian heifers in commercial dairy herds [69], 41% to 53% across semen type and dosage on Holstein heifers [70], 46.8% pregnancy rates in Holstein dairy cows [71], and 46.1% pregnancy rates for Boran and Boran X Holstein cross cows and heifers [72]. The fertility of SS in this trial was significantly greater than that observed in the study by Karakaya et al. [15], who observed a conception rate of 31.8% to 25.7%, and Healy et al. [20], who reported 31.6% conception rates in nulliparous Holstein heifers, 39% mean conception rate for heifers [73], and 21.0% average pregnancy rate for lactating Holstein-Friesian cows [21] using SS. In contrast, higher fertility using SS was also recorded in this study. Correa-Calderón et al. [23] reported a 65% conception rate in winter at Day 35 post-AI in Holstein heifers, 53.9% to 66.3% pregnancy rate using deep intracorneal insemination [74], and 80% (388/485) pregnancy rate in the dairy village production system in Central Ethiopia [75]. The differences observed in the current and previous works could be due to selection criteria used to select the most fertile cows, dam parity and season when AI was performed, semen dosage, insemination techniques used, and overall management system.

## 4. Conclusions

The present study demonstrated that estrus response to a single injection of PGF2α was significantly affected by parity and body condition of the animals while the time interval to onset of estrus was affected by the genetic background of the animals. The pregnancy rate using SS is lower than CS, but it can provide a playground for much needed replacement heifers in the smallholder dairy setting if implemented in the future.

## Figures and Tables

**Table 1:** Estrus response rate of Horro and Holstein-Horro crossed cows and heifers to PGF2α hormone under smallholder farming system in the study area.

Variables	Category	Estrus proportion	%	Odds ratio (95% CI)	*p* value
Parity	Cows	43/60	71.7	Cows (reference)	
	Heifers	53/60	88.3	2.99 (1.13–7.87)	0.026

Breeds	Horro	44/60	73.33	Horro (reference)	
	Cross-breed	52/60	86.67	0.42 (0.16–1.08)	0.073

BCS	Suboptimal	29/46	63.0	Suboptimal (reference)	
	Optimal	67/74	90.5	0.17 (0.06–0.47)	0.001

Total		96/120	80		

**Table 2:** Interval to estrus after PGF2α hormone injection in Horro and Holstein-Horro crossed cows and heifers (mean ± SE) under a smallholder farming system in the study.

Variable	Category	Proportion	%	Time required for the onset of estrus (h)	
Breed	Horro	44/60	73.33	75.41 ± 2.77^a^	*p*=0.0157
	Cross-breed	52/60	86.67	66.0 ± 2.63^b^	

Parity	Heifers	53/60	83.33	71.75 ± 2.3^a^	*p*=0.3409
	Cows	43/60	71.67	67.35 ± 2.19^a^	

BCS	Optimal	67/74	90.54	69.13 ± 1.96^a^	*p*=0.3627
	Suboptimal	29/46	60.03	73.03 ± 3.86^a^	

*Note:* In a column, mean values that are followed by different letters are significantly different at *p* < 0.05 within a group.

Abbreviation: BCS = body condition score.

**Table 3:** Pregnancy rate of PGF2α hormone–treated Horro and Holstein Friesian Horro crossed cows and heifers under a smallholder farming system in the study area.

Factors	Category	Pregnancy proportion	%	Odds ratio (95% CI)	*p* value
Breed	Horro	25/44	56.8	Cross (reference)	
	Cross	33/52	63.46	0.75 (0.33–1.72)	**0.508**

BCS	Suboptimal	16/29	55.1	Suboptimal (reference)	
	Optimal	42/67	62.7	0.73 (0.30–1.77)	**0.490**

Parity	Heifers	30/53	56.6	Heifers (reference)	
	Cows	28/43	65.1	0.69 (0.30–1.60)	**0.397**

Semen type	Conventional	35/48	72.9	Conventional (reference)	
	Sexed	23/48	47.9	2.92 (1.24–6.86)	**0.014**

Total		58/96	60.4		

*Note: p* values are significance for the parameters/variables considered.

## Data Availability

The data that support the findings of this study are available from the corresponding author upon reasonable request.
